# Hypoxia-Inducible Factor-2 Alpha Regulates the Migration of Fibroblast-like Synoviocytes via Oxidative Stress-Induced CD70 Expression in Patients with Rheumatoid Arthritis

**DOI:** 10.3390/ijms23042342

**Published:** 2022-02-20

**Authors:** Su-Jin Yoo, Ha-Reum Lee, Jinhyun Kim, In Seol Yoo, Chan Keol Park, Seong Wook Kang

**Affiliations:** 1 Division of Rheumatology, Department of Internal Medicine, Chungnam National University Hospital, 282 Munhwaro, Daejeon 35015, Korea; sujin428@cnuh.co.kr (S.-J.Y.); hareum_lee@cnu.ac.kr (H.-R.L.); jkim@cnuh.co.kr (J.K.); 2 Research Institute for Medical Sciences, Chungnam National University School of Medicine, 266 Munhwaro, Daejeon 35015, Korea; 3 Division of Rheumatology, Department of Internal Medicine, Chungnam National University Sejong Hospital, 20 Bodeum-7-ro, Sejong 30099, Korea; cptmiller@cnuh.co.kr (I.S.Y.); plutocys@cnuh.co.kr (C.K.P.)

**Keywords:** rheumatoid arthritis, reactive oxygen species, cytokines, antioxidants, synovial fluid

## Abstract

This study aimed to examine the role of CD70, which is highly expressed on fibroblast-like synoviocytes (FLS), in rheumatoid arthritis (RA) patients. FLS isolated from RA (*n* = 14) and osteoarthritis (OA, *n* = 4) patients were stimulated with recombinant interleukin-17 (IL-17; 5 ng/mL) and tumor necrosis factor alpha (TNF-α; 5 ng/mL) for 24 h. Expression of CD70, CD27/soluble CD27 (sCD27), and hypoxia-inducible factor-2 alpha (HIF-2α) was analyzed by RT-qPCR, flow cytometry, and ELISA assays, respectively. Reactive oxygen species (ROS) expression and cell migration were also examined. The HIF-2α inhibitor PT-2385 and CD70 inhibitor BU69 were used to specifically suppress these pathways. Stimulation with IL-17 and TNF-α significantly induced CD70 expression in RA FLS. Although the synovial fluids from patients with RA contained high levels of sCD27, surface expression of CD27, a ligand of CD70, was rarely detected in RA FLS. Cytokine-induced CD70 expression was significantly decreased following antioxidant treatment. Following HIF-2α inhibition, RA FLS had decreased expression of CD70 and ROS levels. Migration of RA FLS was also inhibited by inhibition of CD70 or HIF-2α. The surface expression of CD70 is regulated by HIF-2α and ROS levels and is a key contributor to cytokine-enhanced migration in RA FLS.

## 1. Introduction

Rheumatoid arthritis (RA) is a progressive chronic inflammatory autoimmune disease characterized by synovial hyperplasia, pannus formation, synovium inflammation, and bone destruction [1,2]. The pathogenesis of RA has been characterized as an excessive infiltration of immune cells into synovial joints, stimulating the migration of fibroblast-like synoviocytes (FLS) into unaffected joints, encouraging excessive oxidative stress in synovium [3,4,5]. Recently, we reported that reactive oxygen species (ROS) are critical factors for vascular cell adhesion protein 1 (VCAM1)—vascular endothelial growth factor (VEGF)-migration signaling in RA FLS [6]. Although ROS levels and the migratory ability of RA FLS are considered important contributors to disease severity, the cause of RA remains unclear.

CD70 is a member of the tumor necrosis factor (TNF) superfamily and a ligand for CD27 [7,8,9]. CD70, mainly expressed on T cells, B cells, and tumor cells, leads to activation of immune cells [10,11]. CD70/CD27 signaling is a primarily regulated by the expression of CD70 and can directly regulate T-cell–T-cell interactions and influence the development of effector T cells [12]. Therefore, high expression of CD70 directly induces excessive infiltration of CD27^+^ lymphocytes and is associated with hematologic malignancies [13,14]. Additionally, CD27 and CD70 interactions produce soluble CD27 (sCD27) [15]. A high concentration of sCD27 in the sera is a marker of poor prognosis in T-cell lymphoma [16]. Additionally, the level of sCD27 is higher in sera from patients with juvenile idiopathic arthritis compared to healthy controls [17]. CD70-expressing T cells are more enriched in the peripheral blood of patients with RA than that of healthy controls and are correlated with increased production of interferon (IFN)-γ and interleukin (IL)-17 [18,19]. However, the expression and role of CD70 is still unknown in FLS from patients with RA.

The inflamed synovium in RA is generally induced by low-oxygen conditions (hypoxia) in proliferating FLS, accumulation of inflammatory cells, and formation of angiogenesis [20,21]. Hypoxia-inducible factor-2 alpha (HIF-2α) is a transcriptional factor that acts as an essential catabolic regulator in the hypoxic and inflamed synovium [22]. The expression of HIF-2α is up-regulated in the intimal lining of the human RA synovium [23]. In cancer, HIF-2α enhances CD70 expression, which mediates tumor progression and aggressiveness and is associated with poor prognosis [24]. In this study, we investigated the role of CD70 in FLS migration and the correlation with HIF-2α expression in patients with RA. We found that the surface expression of CD70 is modulated by HIF-2α-mediated ROS levels, and RA FLS is controlled by HIF-2α/ROS/CD70-mediated signaling. These findings suggest a novel approach to suppress the initiation and development of RA through ROS regulation in FLS.

## 2. Results

### 2.1. IL-17- and TNF-α-Induced CD70 Expression in RA FLS

**Figure 1:**
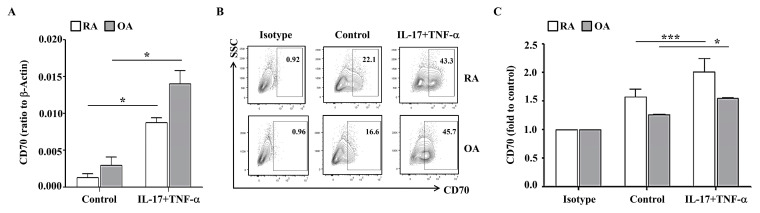
IL-17 and TNF-α stimulate CD70 expression in RA FLS and OA FLS. (**A**) RA FLS (*n* = 3; white bar) and OA FLS (*n* = 4; grey bar) were cultured with or without IL-17 (5 ng/mL) and TNF-α (5 ng/mL) for 24 h. CD70 mRNA levels were quantified using RT-PCR and β-actin as a housekeeping gene. Statistical differences between groups were determined using a Mann–Whitney test. (**B**) RA FLS and OA FLS were stimulated with or without IL-17 (5 ng/mL) and TNF-α (5 ng/mL) for 24 h. (**C**) RA FLS (*n* = 14; white bar) and OA FLS (*n* = 4; grey bar) were stimulated with or without IL-17 (5 ng/mL) and TNF-α (5 ng/mL) for 24 h. To analyze the surface expressions of CD70, flow cytometry was performed using PE-conjugated anti-CD70. Average CD70 expression is displayed as fold change of the mean fluorescence intensity (MFI) compared to control. Data are presented as mean ± standard deviation. Statistical analysis was performed by using a Mann–Whitney test (RA) or a paired *t*-test (OA). * indicates *p* < 0.05. *** indicates *p* < 0.001.

The surface expression of CD70 on RA FLS is still unknown. We first examined the level of CD70 in RA FLS and OA FLS. For induction of pro-inflammatory conditions, FLS were stimulated with recombinant human IL-17 and TNF-a, which were detected with higher levels in synovial fluid from the patients with RA than OA [25]. *CD70* mRNA levels were significantly increased by cytokine treatment in both RA FLS (0.009 ± 0.001 versus no treatment 0.001 ± 0.001; *n* = 3) and OA FLS (0.014 ± 0.002 versus no treatment 0.003 ± 0.001; *n* = 4) (Figure 1A). The intrinsic surface expression of CD70 was also determined in RA FLS and OA FLS (Figure 1B). RA FLS (2.003 ± 0.247; *n* = 14) had higher cytokine-induced CD70 expression than OA FLS (1.560 ± 0.002; *n* = 4) (Figure 1C). These data indicate that RA FLS had increased surface expression of CD70 in response to IL-17 and TNF-α stimulation in comparison to OA FLS.

### 2.2. CD27 Is Difficult to Detect in RA FLS

**Figure 2:**
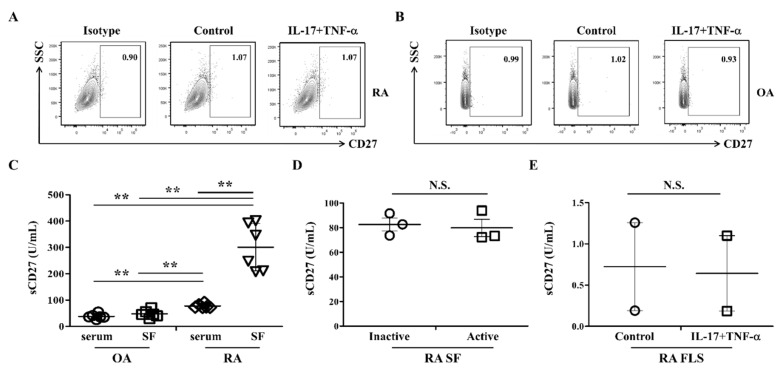
CD27 and sCD27 expression in RA FLS and OA FLS are unchanged by IL-17 and TNF-α stimulation. (**A**) RA FLS and (**B**) OA FLS were stimulated with or without IL-17 (5 ng/mL) and TNF-α (5 ng/mL) for 24 h. The surface expression of CD27 was stained using PerCP-Cy5.5-conjugated anti-CD27. Experiments were performed in triplicate; data from one representative experiment are presented. (**C**) Levels of secreted sCD27 were measured in serum and synovial fluids (SF) from patients with RA and OA (*n* = 6 per group). Statistical differences between groups were determined using a Mann–Whitney test. (**D**) RA activity was categorized according to the Disease Activity Score 28 (DAS28): active (DAS28 > 5.1; 3 patients) and inactive (DAS28 < 2.6; 3 patients). Levels of secreted sCD27 were measured in synovial fluids (SF) these subgroups (*n* = 3 per group). (**E**) RA FLS (*n* = 2) were stimulated with or without IL-17 (5 ng/mL) and TNF-α (5 ng/mL). After 24 h, culture supernatants were analyzed by ELISA to measure secreted levels of sCD27. ** indicates *p* < 0.01. NS, not significant.

Next, we determined the surface expression of CD27, which is a ligand for CD70 [8]. The intrinsic expression of CD27 was not detected in RA FLS or OA FLS (Figure 2A,B). Even after stimulation with IL-17 and TNF-α, CD27 expression did not change from the control. The production of sCD27 was accessed using sera and synovial fluids. Patients with RA had the higher expression of sCD27 than patients with OA (*n* = 6 per group; Figure 2C). When sCD27 expression was analyzed in paired samples of sera and synovial fluids from patients with RA, synovial fluids had higher sCD27 levels than sera. There were no differences between the two groups of patients based on RA severity (*n* = 3 per group; Figure 2D). Treatment with IL-17 and TNF-α did not significantly increase levels of sCD27 in RA FLS (*n* = 2 per group; Figure 2E). Taken together, these results suggest that sCD27 in synovial fluids from patients with RA may be produced from immune cells, such as T cells. We also showed that RA FLS expressed CD70, but CD27 was not detectable.

### 2.3. CD70 Expression Is Regulated by ROS in RA FLS

**Figure 3:**
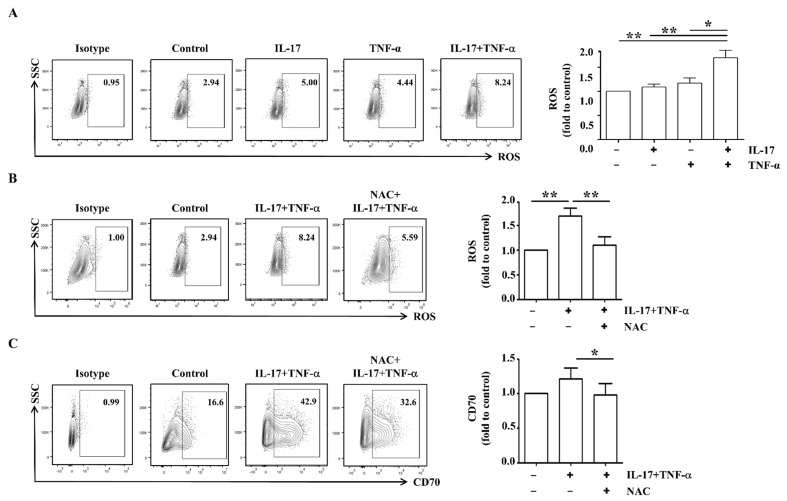
IL-17 and TNF-α enhance ROS expression in RA FLS. (**A**) RA FLS (*n* = 8) were cultured with or without IL-17 (5 ng/mL) and TNF-α (5 ng/mL) for 24 h. To analyze ROS expression, flow cytometry analysis was performed using DCFDA. Data from one representative experiment are presented (left). Average ROS expression was displayed as fold change of the MFI compared to control (right). (**B**) RA FLS (*n* = 8) were pre-incubated with 10 nM of NAC for 1 h, and then, cells were stimulated with or without IL-17 (5 ng/mL) and TNF-α (5 ng/mL) for 24 h. To analyze ROS expression, flow cytometry analysis was performed using DCFDA. Data from one representative experiment are presented (left). Average ROS expression is displayed as fold change of the MFI compared to control (right). (**C**) RA FLS (*n* = 5) were pre-incubated with 10 nM of NAC for 1 h, and then, cells were stimulated with or without IL-17 (5 ng/mL) and TNF-α (5 ng/mL) for 24 h. To analyze the surface expressions of CD70, flow cytometry analysis was performed using PE-conjugated anti-CD70. Data from one representative experiment are presented (left). Average CD70 expression was calculated as fold change of the MFI compared to control (right). Statistical analysis was performed using a Mann–Whitney test. * indicates *p* < 0.05; ** indicates *p* < 0.01.

Synovial fluids in patients with RA contain many inflammatory cells and ROS, which mediate disease severity [26]. We investigated whether IL-17 and TNF-α induce cellular ROS expression in FLS from patients with RA. Intracellular ROS levels in RA FLS were elevated by 1.7-fold after stimulation with cytokines compared to control (*n* = 8; Figure 3A). When RA FLS were pre-treated with NAC for 1 h, ROS levels were significantly reduced by 35.3% in RA FLS stimulated with cytokines compared to those without NAC (*n* = 8; Figure 3B). Furthermore, surface expression of CD70 was decreased by 19.1% after treatment with both NAC and cytokines compared to treatment with cytokines alone (*n* = 5; Figure 3C). These data suggest that ROS may induce the increase in surface expression of CD70 in RA FLS.

### 2.4. HIF-2α Inhibition Decreased CD70 Expression in RA FLS

**Figure 4:**
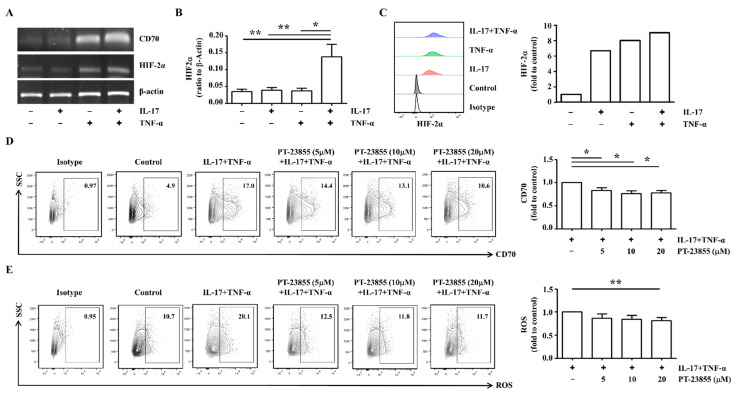
CD70 expression in RA FLS is downregulated by HIF-2α-specific inhibition. (**A**) RA FLS were cultured with or without IL-17 (5 ng/mL) and TNF-α (5 ng/mL) for 24 h. (**B**) RA FLS (*n* = 6) were cultured with or without IL-17 (5 ng/mL) and TNF-α (5 ng/mL) for 24 h. mRNA levels of CD70 or HIF-2α were assessed by RT-PCR, using β-actin as the housekeeping gene. Statistical differences were determined using one-way ANOVA. (**C**) To analyze the intracellular expressions of HIF-2α, RA FLS were cultured with or without IL-17 (5 ng/mL) and TNF-α (5 ng/mL) for 24 h. Following fixation and permeabilization, RA FLS were stained using rabbit anti-human HIF-2α with FITC-conjugated anti-rabbit IgG secondary antibody. Data from one representative experiment are presented (left). The averages of HIF-2α expression are displayed as fold change of the MFI compared to control (right). (**D**) RA FLS (*n* = 4) were cultured with or without IL-17 (5 ng/mL) and TNF-α (5 ng/mL) for 24 h. To inhibit HIF-2α, cells were co-treated with 5, 10, or 20 μM of PT-2385 for 24 h. To analyze surface expression of CD70, flow cytometry analysis was performed using PE-conjugated anti-CD70. Data from one representative experiment are presented (left). The averages of CD70 expression are displayed as fold change of the MFI compared to cytokine-stimulated cells (right). Statistical analysis was performed using a Mann–Whitney test. (**E**) RA FLS (*n* = 5) were cultured with or without IL-17 (5 ng/mL) and TNF-α (5 ng/mL) for 24 h. To inhibit HIF-2α, cells were co-treated with 5, 10, and 20 μM of PT-2385 for 24 h. To analyze ROS levels, flow cytometry was performed using DCFDA. Data from one representative experiment are presented (left). Average ROS levels are displayed as fold change of the MFI compared to cytokine-stimulated cells (right). Statistical analysis was performed using a Mann–Whitney test. The bar represents the mean. * indicates *p* < 0.05; ** indicates *p* < 0.01.

HIF-2α is a representative key protein regulating the cellular response to hypoxia [22]. When RA FLS were incubated with IL-17 and TNF-α, mRNA expression of HIF-2α was synergistically elevated in IL-17/TNF-α-treated cells (0.035 ± 0.006) compared unstimulated control FLS (0.137 ± 0.036) (*n* = 6; Figure 4A,B). HIF-2α protein levels were also markedly elevated by 9.01-fold in cytokine stimulation compared to control (Figure 4C). To determine whether HIF-2α influenced CD70, HIF-2α was inhibited by PT-2385 treatment. The cytokine-induced CD70 levels were significantly decreased by 21.9% following 20 uM of HIF-2α inhibitor treatment (*n* = 4; Figure 4D). ROS levels were also significantly downregulated by 18.7% following 20 uM of HIF-2α inhibitor treatment (*n* = 5; Figure 4E). These results suggest that HIF-2α modulates the surface expression of CD70 in RA FLS through ROS regulation.

### 2.5. Enhanced Expression of CD70 Is Associated with Increased Migration in RS FLS

**Figure 5:**
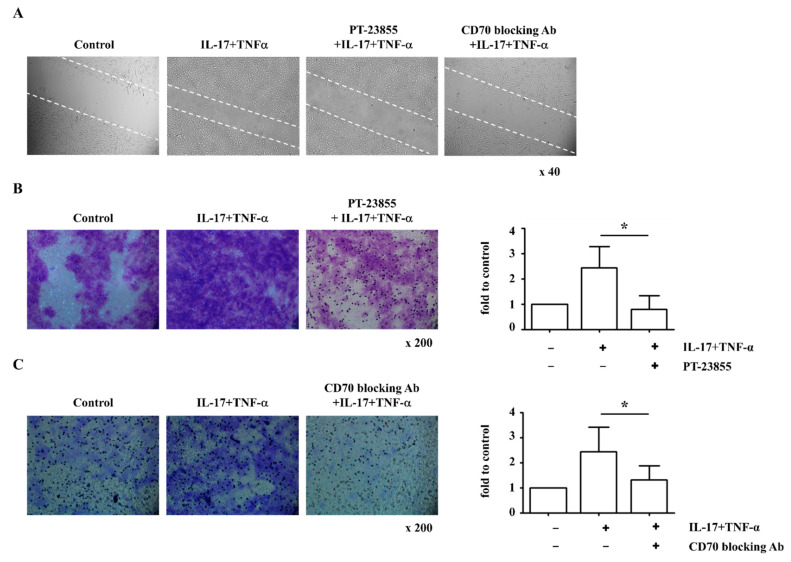
IL-17- and TNF-α-induced migration is attenuated by inhibiting CD70 or HIF-2α. (**A**) RA FLS were cultured with or without IL-17 (5 ng/mL) and TNF-α (5 ng/mL) for 24 h. To inhibit HIF-2α, 20 μM PT-2385 was added to the culture for 24 h. To inhibit CD70, 1 μg/mL BU69 was added to the culture for 24 h. Following the scratch assay, migrated cells were photographed after cytokine stimulation for 24 h. Magnification is 40×. (**B**,**C**) Cell migration of RA FLS (*n* = 4) was measured using a transwell chamber after 16 h and visualized with crystal violet staining. Following solubilization, the crystal violet dye was quantitated using optical density. Data represent the fold change of the optical density compared to control. Statistical analysis was performed using a Mann–Whitney test. Magnification is 200×. * indicates *p* < 0.05.

Migration of activated FLS mediates bone damage in RA progression via invasion through the cartilage [27]. We therefore investigated whether RA FLS cell migration is associated with increased CD70 expression. Stimulation with IL-17 and TNF-α enhanced cell movement into the wounded area, and cytokine-induced cell movement was decreased by PT-2385 (HIF-2α inhibitor) or BU69 (CD70-blocking antibody) (Figure 5A). When cell migration was analyzed using a transwell membrane, IL-17 and TNF-α stimulation also enhanced the migratory ability of RA FLS compared to unstimulated RA FLS controls, and inhibition of HIF-2α (33.0% less) and CD70 (29.4% less) attenuated cell migration (*n* = 5; Figure 5B,C). Based on these results, we suggest that CD70 influences cell migration via regulation of HIF-2α and ROS in RA FLS.

## 3. Discussion

RA FLS were stimulated with IL-17 and TNF-α, which are regarded as key cytokines in RA pathogenesis but not in OA [28,29]. These cytokines are detected in RA synovium, and their inhibition is a therapeutic target for RA treatments [30]. Here, IL-17 and TNF-α significantly enhanced the expression of CD70 in RA FLS compared to OA FLS (Figure 1). This is the first study, to our knowledge, to report the presence of CD70 in RA FLS. Surface expression of CD70 has been associated with a higher risk of cancer malignancy and aberrant T-cell activation [13] in part due to CD70/CD27 interactions that can induce excessive infiltration of T cells and cause autoimmune disease [18,19]. Although CD27 was not detected on the surface of RA FLS, sCD27 was detected in synovial fluid from patients with RA (Figure 2). These results suggest that the high level of sCD27 could originate from activated T cells through interactions with CD70 expressing FLS in RA. Subsequently, CD70 could act as an important co-stimulatory molecule in the development of RA. Furthermore, the difference in expression of sCD27 between RA and OA in synovial fluids could be a potential diagnostic marker for RA or an active inflammation state.

CD70 expression is modulated by ROS levels [31]. In RA FLS, surface expression of CD70 was also inhibited by pretreatment with antioxidants (Figure 3C). Production of ROS is increased in patients with RA relative to healthy controls due to an insufficient antioxidant defense system [32,33]. Oxidative stress leads to the generation of deleterious byproducts that mediate cell toxicity, the production of pro-inflammatory cytokines, and ultimately damage to structural and functional cartilage [33]. Several transcription factors, including nuclear factor kappa-light-chain-enhancer of activated B cells, activating protein-1, p53, HIF-1α, and HIF-2α, have been shown to be redox-sensitive [34,35]. Following stimulation with IL-17 and TNF-α, mRNA and protein levels of HIF-2α were elevated in RA FLS (Figure 4A–C). After HIF-2α-specific inhibition in RA FLS, the surface expression of CD70 and ROS levels were significantly reduced (Figure 4D,E). Although the correlation between ROS and CD70 has been suggested as an important signaling pathway in RA, few studies have investigated the relationship between HIF-2α and CD70. These results suggest IL-17 and TNF-α-mediated HIF-2α expression activates ROS-CD70 signaling.

FLS in the synovial intimal lining are the major cause of cartilage destruction due to migration to the unaffected joints in RA [36]. The active migratory phenotype and strong cartilage invasiveness are unique characters of RA FLS [37]. Previous studies showed that CD70-expressing cancer-associated fibroblasts showed increased migratory capacities [38], and inhibition of CD70 in primary glioblastoma suppressed tumor migration [39]. In mice, HIF-2α was also regulated by stimulating FLS migration and invasion, leading to cartilage erosion during RA pathogenesis [40]. We showed that inhibition of HIF-2α or CD70 attenuated the migratory ability of RA FLS using wound and transwell migration assays (Figure 5). From these results, we suggest that CD70 influences FLS migration via regulation of HIF-2α and ROS in RA. In a collagen-induced arthritis model, treatment with CD70-blocking antibody resulted in marked improvements in disease severity and a significant reduction in the production of autoantibodies [41]. Further studies are needed to analyze the suppressive function of T cells in synovial fluid with CD70-decreased RA FLS. Our findings suggest HIF-2α/ROS/CD70 signaling could be a novel therapeutic strategy for RA treatment and may contribute to a better understanding of RA initiation and progression.

## 4. Materials and Methods

### 4.1. Human Subjects

Whole blood and synovial fluid samples were collected from patients with RA (*n* = 6) and osteoarthritis (OA, *n* = 6) using heparin tubes at Chungnam National University Hospital (Daejeon, Korea). Patients were diagnosed with RA according to the American College of Rheumatology (ACR)/European League Against Rheumatism (EULAR) 2010 classification criteria [42]. RA patients were excluded as a result of any of the following disorders: infectious disease, overlap of autoimmune conditions suspected, septic arthritis, osteoarthritis, lupus, cancer, multiple sclerosis, and stroke. Patients with OA were diagnosed according to the 1985 ACR criteria [43]. OA patients were excluded in the event of any of the following disorders: inflammatory diseases, such as septic arthritis and rheumatoid arthritis; history of knee surgery, infectious diseases, cancer, multiple sclerosis, stroke, and immune system disorder. Synovial fluid samples were obtained from knee arthrocentesis, which was performed for diagnosis or treatment. Sera and synovial fluids were centrifuged, and the supernatant was stored at −80 °C. Synovial tissues were obtained from patients with RA (*n* = 14; Table 1) and OA (*n* = 4) who had undergone synovectomy or joint replacement. After removing fat and fibrous tissues, the synovium was cut into small pieces and incubated with 0.1% collagenase (Sigma-Aldrich, MO, USA) in Dulbecco’s modified Eagle’s medium (DMEM; Gibco, MA, USA) at 37 °C for 2 h. Cells were cultured in DMEM supplemented with 10% fetal bovine serum (FBS; Gibco), 100 U/mL penicillin, and 100 mg/mL streptomycin and maintained in a 5% CO_2_ incubator at 37 °C. The purity of isolated FLS (CD90^+^) was determined to be >95% (data not shown). FLS were used for experiments after three to six passages.

### 4.2. Ethics Statement

This study was performed according to the recommendations of the Declaration of Helsinki and approved by the Institutional Review Board of Chungnam National University Hospital (2017-12-024-002). All study patients signed informed written consent before participation.

### 4.3. Real-Time PCR and RT-PCR

Total RNA was extracted using TRI Reagent (Molecular Research Center, OH, USA), according to the manufacturer’s instructions. Extracted RNA was used in reverse transcription reactions with ReverTra Ace^®^ qPCR RT Master Mix (TOYOBO, Osaka, Japan), according to the manufacturer’s instructions. SYBR^®^ Green Realtime PCR Master Mix (TOYOBO) was used for RT-PCR analysis of cDNA following the manufacturer’s instructions. The primers were synthesized by Bioneer (Daejoen, Korea; see Table 2 for primer sequences). Thermal cycling conditions were as follows: initial denaturation at 95 °C for 5 min, 40 cycles of 95 °C for 10 s, 60 °C for 15 s, and 72 °C for 20 s. A melting step was performed by raising the temperature from 72 °C to 95 °C after the last cycle. Thermal cycling was conducted using a CFX Connect RT-PCR Detection System (Bio-Rad Laboratories, Inc., Hercules, CA, USA). The target gene expression levels are shown as a ratio in comparison with the levels of β-actin in the same sample via calculation of the cycle threshold (Ct) value. The relative expression levels of target genes were calculated by the 2^−ΔΔCT^ relative quantification method.

For RT-PCR, the synthesized cDNA was mixed with Solg™ 2X Taq PCR Pre-Mix (SolGent, Daejoen, Korea) and 10 pmol of each specific PCR primer, as specified in the manufacturer’s protocol. Amplified products were separated on 2% agarose gels, stained with Midori green advance (NIPPON Genetics EUROPE, Düren, Germany), and photographed under UV illumination using a GelDoc system (Bio-Rad Laboratories, Inc.).

### 4.4. Flow Cytometry Analysis

To analyze the surface expression of CD70 or CD27, FLS were stained using phycoerythrin (PE)-conjugated anti-CD70 (cat# 555835; BD Biosciences, NJ, USA) and PerCP-Cy5.5-conjugated anti-CD27 (cat# 560612; BD Biosciences). Intracellular expression of HIF-2α was detected using rabbit anti-human HIF-2α (cat# NB100-122; NovusBio, CO, USA) with FITC-conjugated anti-rabbit IgG secondary antibody (cat# ab150077; Abcam, Cambridge, UK).

To detect ROS levels, cells were stained with DCFDA (cat# C6827; Invitrogen, MA, USA), according to the manufacturer’s instructions. The antioxidant, *N*-acetyl-l-cysteine (NAC, cat# A7250), was obtained from Sigma-Aldrich. Cells were analyzed with a FACSCanto II flow cytometer (BD Biosciences), and data were processed with FlowJo software (Tree Star, OR, USA).

### 4.5. Enzyme-Linked Immunosorbent Assay (ELISA)

An ELISA kit for human soluble CD27 (Invitrogen) was used to measure sCD27 according to the manufacturer’s instructions, and sCD27 levels were estimated by interpolation from a standard curve generated using a Sunrise absorbance reader (Tecan, Männedorf, Switzerland) at 450 nm. Data were analyzed using XFLUOR4 (Tecan, Version 4.51).

### 4.6. Wound Migration Assay

When FLS cultures were approximately 90% confluent, cells were incubated with DMEM supplemented with 1% FBS for 4 h. FLS monolayers were wounded with pipette tips and treated for 24 h with recombinant human IL-17 (PeproTech, Cranbury, NJ, USA) and TNF-α (PeproTech). During the blocking assays, the cultures were treated with HIF-2α inhibitor (PT-2385; Abcam) or CD70-blocking antibody (BU69; Abcam). Wound closure was monitored and photographed at 0 and 24 h with an Olympus inverted microscope (magnification 40×; 0.55 numerical aperture dry objective; Tokyo, Japan). To quantify the migrated cells, pictures of the initial wounded monolayers were compared with the corresponding pictures of cells at the end of the incubation.

### 4.7. Transwell Migration Assay

RA FLS were stimulated with recombinant IL-17 (5 ng/mL, PeproTech) and TNF-α (5 ng/mL, PeproTech) for 24 h. For the transwell migration assay, cells were centrifuged and loaded onto the top of transwell filters with an 8-μm pore opening (Corning, Inc., New York, NY, USA). DMEM containing 10% FBS was transferred to the bottom chamber of the transwell plate as a chemoattractant for 16 h. Then, transwells were fixed with 100% methanol and stained with dilute crystal violet (Sigma-Aldrich). Non-migrating cells on the top membrane surface were removed by washing with PBS and cotton swabs. Invaded cells were manually counted in five random fields per sample under an inverted microscope (magnification 200×; 0.55 numerical aperture dry objective; Olympus). The crystal violet dye was diluted with 0.1% sodium dodecyl sulfate (SDS; ELPIS biotech, Daejoen, Korea). The number of cells marked with crystal violet was quantified using the optical density and a Sunrise absorbance reader (Tecan) at 595 nm.

### 4.8. Statistical Analysis

Results are expressed as the mean ± standard deviation. Data were first tested for conformation to a normal distribution. For comparison between two groups, Student’s *t*-test or nonparametric Mann–Whitney tests were applied depending on the data distribution. For comparison between three or more groups, one-way analysis of variance (ANOVA) test or nonparametric Kruskal–Wallis test was applied. *p*-Values < 0.05 were considered statistically significant. All analyses were performed using SPSS version 26 (IBM, Armonk, NY, USA).

## Figures and Tables

**Table 1:** Baseline characteristics of all patients.

VARIABLES		RA (*n* = 41)	OA (*n* = 4)
Female (*n*, %)		10 (71.4%)	3 (75.0%)
Age (year, mean ± SD)		63.86 ± 1.77	70.0 ± 6.39
BMI (Kg/m^2^ ± SD)		23.78 ± 0.62	27.79 ± 1.32
Laboratory features (*n*, %)	Antinuclear antibody (ANA)	4 (28.6%)	-
	Rheumatoid factor (RF)	13 (92.9%)	-
	Anti CCP Ab	11 (78.9%)	-
Treatment (*n*, %)	Methotrexate	11 (78.9%)	-
	Hydroxychloroquine	8 (57.1%)	-
	Sulfasalazine	6 (42.9%)	-
	Leflunomide	2 (14.3%)	-
	Tacrolimus	1 (7.1%)	-
	Steroid	11 (78.9%)	-

SD, standard deviation.

**Table 2:** Primers used for PCR.

	Sense Primer	Antisense Primer
*CD70*	TCTCAGCTTCCACCAAGGTT	AAGTGTCCCAGTGAGGTTGG
*HIF-2α*	CCTTAAGACAAGGTCTGCA	TTCATCCGTTTCCACATCAA
*β-actin*	ACAATGAGCTGCTGGTGGCT	TGGGCACAGTGTGGGTGA

## Data Availability

Not applicable.
